# Seasonal Molecular Difference in Fibrillar Collagen Extracts Derived from the Marine Sponge *Chondrosia reniformis* (Nardo, 1847) and Their Impact on Its Derived Biomaterials

**DOI:** 10.3390/md21040210

**Published:** 2023-03-28

**Authors:** Eleonora Tassara, Boaz Orel, Micha Ilan, Dario Cavallo, Andrea Dodero, Maila Castellano, Silvia Vicini, Marco Giovine, Marina Pozzolini

**Affiliations:** 1 Department of Earth, Environment and Life Sciences (DISTAV), University of Genova, Via Pastore 3, 16132 Genova, Italy; 2 George S. Wise Faculty of Life Sciences, School of Zoology, Tel Aviv University, Tel Aviv 6997801, Israel; 3 Department of Chemistry and Industrial Chemistry (DCCI), University of Genova, Via Dodecaneso 31, 16146 Genova, Italy; 4 Adolphe Merkle Institute (AMI), University of Fribourg, Chemin des Verdiers 4, 1700 Fribourg, Switzerland

**Keywords:** Porifera, demosponges, biomaterial, collagen

## Abstract

*Chondrosia reniformis* (Nardo, 1847) is a marine sponge of high biotechnological interest both for its natural compound content and for its peculiar collagen, which is suitable for the production of innovative biomaterials in the form, for instance, of 2D membranes and hydrogels, exploitable in the fields of tissue engineering and regenerative medicine. In this study, the molecular and chemical-physical properties of fibrillar collagen extracted from specimens collected in different seasons are studied to evaluate the possible impact of sea temperature on them. Collagen fibrils were extracted from sponges harvested by the Sdot Yam coast (Israel) during winter (sea temperature: 17 °C) and during summer (sea temperature: 27 °C). The total AA composition of the two different collagens was evaluated, together with their thermal stability and glycosylation level. The results showed a lower lysyl-hydroxylation level, lower thermal stability, and lower protein glycosylation level in fibrils extracted from 17 °C animals compared to those from 27 °C animals, while no differences were noticed in the GAGs content. Membranes obtained with fibrils deriving from 17 °C samples showed a higher stiffness if compared to the 27 °C ones. The lower mechanical properties shown by 27 °C fibrils are suggestive of some unknown molecular changes in collagen fibrils, perhaps related to the creeping behavior of *C. reniformis* during summer. Overall, the differences in collagen properties gain relevance as they can guide the intended use of the biomaterial.

## 1. Introduction

For a few decades, marine collagen has been considered a high-value and promising alternative biopolymer to those derived from mammals for many biomedical application purposes. There are many reasons for this growing interest in marine collagens. Specifically, the risk of BSE (bovine spongiform encephalopathy), TSE (transmissible spongiform encephalopathy), and religious constraints (e.g., avoidance of porcine derivatives) are now considered relevant problems for the use of collagens derived from mammals. In this context, marine collagen is now proposed as a valid solution [1]. The scientific literature is rich in publications giving many examples of potential applications of fish and marine invertebrates’ collagens for tissue engineering and regenerative medicine [2,3,4,5]. In the creation of collagen-based biomaterials for regenerative medicine, besides the mechanical properties, particular attention is paid to the thermal stability of the collagen, which should be compatible with the human body temperature. One of the main limitations of collagens of marine origin, typically isolated from fish or invertebrates, is their reduced thermal stability if compared with that of terrestrial homeotherms [6]. In these circumstances, the strong interest in finding new molecular types of marine collagens having the correct characteristics for their application in the biomedical field becomes evident.

The collagen molecules are characteristically assembled in a typical right-handed a-helical structure of three identical (homotrimer) or different (heterotrimer) α-chains, bound together to form a triple helix. This structural domain is a common denominator, but among animals, many collagen types exist due to the different specific roles they have in the complex structural organization of the extracellular matrix. Many diversified isoforms of collagen can be found in nature, not only in humans and other mammals (for which more than 20 different collagen types are known [7]) but in simpler animals as well. In the marine world, in addition to the fibrillar collagens diffused in all phyla, has been described a large number of type IV-like short chains collagens in sponges and other invertebrates [8] and the very unusual collagen of echinoderms that determines the stiffening and relaxation of the animal body [1,9,10].

The variety of collagens’ properties depends on two aspects: genetic differences and post-translational modifications. The former is related to the evolutionary pressure [11] and to the animals’ specific adaptation to their living environment; the latter are instead linked to different moments of the life cycle [12] and to seasonal changes in the environmental parameters, such as temperature [13]. Animals can express different collagen genes at different times and in different tissues [14], and in addition, some collagen molecules can have various levels of post-translational modifications related to developmental stages or seasonality [13]. The most known modifications are the hydroxylation of proline and lysine, but protein glycosylation and phosphorylation phenomena can also occur [15,16]. The hydroxyproline content plays a role in stabilizing the collagen’s triple helix through a stereo-electronic effect [17], and, according to the literature, it is considered one of the fundamental contributors to the thermal stability of collagens [18]. The thermal stability of collagens is also related, among other things, to the environmental temperature in which an animal lives [13]; that is the reason why this parameter is usually higher in mammalians than in marine organisms [19]. Intermolecular cross-linking between single polypeptides is fundamental to guarantee the mechanical functions of collagens. This condition is the consequence of post-translational modifications occurring during collagen biosynthesis, including the conversion of specific lysine and hydroxylysine residues to the respective aldehydes by lysyl oxidases [20]. These chemical modifications are the beginning of a process that triggers a series of condensation reactions among the lysine derivatives and histidine residues within the same and neighboring molecules to form di, tri-, and tetravalent cross-links [21].

Due to its chemical-physical features, in the last years, it has been demonstrated that collagen derived from the demosponge *Chondrosia reniformis* is useful for biotechnological purposes. *C. reniformis*’ collagen-derived nanoparticles can be used in transdermal drug delivery [22], while in the form of hydrolyzed peptides, it showed interesting anti-photo aging and antioxidant properties [23]. When intact collagen fibrillar extract in combination with glycosaminoglycans (GAGs) component is isolated from the *C. reniformis* sponge tissues, it can be successfully used to produce 2-D membranes that show remarkable antioxidant and biocompatibility properties, with potential application in biomedicine [3]. Considering this broad biotechnological interest, numerous studies have focused on optimizing collagen extraction processes [24] and aquaculture procedures to obtain the large biomass of this sponge [25,26,27]. Moreover, *C. reniformis* can also be found in very shallow waters, subjected to wide temperature variations during the year. Therefore, we hypothesized that this animal could be able to fit into these environmental conditions thanks also to biochemical mechanisms of adaptation involving the collagen itself.

To assess this and to consequently verify the optimal conditions of sponge collection in future aquaculture implants for the production of high-performing collagen, in this work, we evaluated the thermal properties and the biochemical differences of the fibrillar collagen extract of *C. reniformis* specimens collected in summer and in winter and tested their impact on the production of collagen-based devices for biomedical applications. For this purpose, collagen fibrils were extracted from *C. reniformis* specimens collected in the area of the Sdot Yam coast (Israel) in two different periods, during winter (with a sea temperature of 17 °C) and during summer (with a sea temperature of 27 °C). The total AA composition of collagens extracted was evaluated, together with the glycosylation and GAGs level and their thermal stability and viscosity. Finally, from the two different types of collagen extract, 2-D membranes were produced, and their stiffness and dynamic-thermal stability, as well as their antioxidant and biocompatibility properties, were compared.

## 2. Results and Discussion

### 2.1. Biochemical Characterization of Sponge Fibrillar Collagen Extracts

#### 2.1.1. Evaluation of Amino-Acids Percentage Composition

The fibrillar collagen suspension was isolated from 17 °C and 27 °C sponges according to the procedures described in the methods section, and the total amino acid composition of the two extracts was evaluated. The results are summarized in Table 1, which presents a substantial similarity between the amino acid profiles in the two samples.

**Figure 1:**
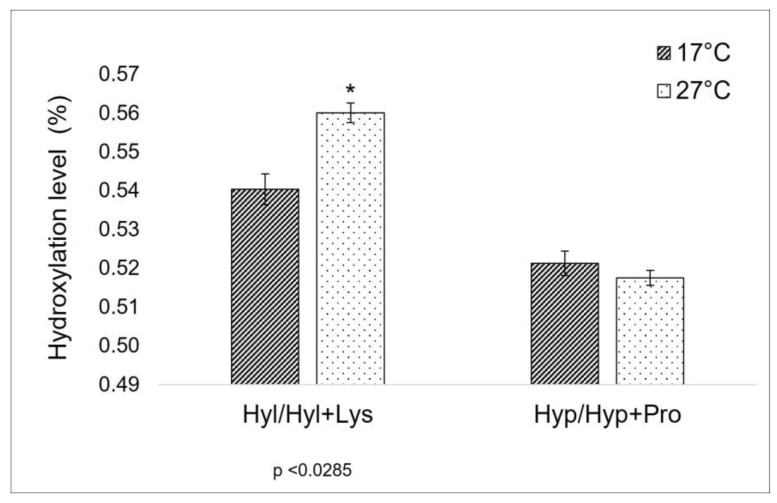
Hydroxylation ratio in collagen extracted from sponges collected during cold (17 °C) and warm (27 °C) seasons. Hyl/Hyl + Lys: the ratio between hydroxylysine and hydroxylysine + lysine amount (values from Table 1); Hyp/Hyp + Pro: the ratio between hydroxyproline and hydroxyproline+ proline amount (values from Table 1). Asterisks indicate a significant difference from the respective control (paired Tukey test, * *p* < 0.05).

As previously explained in the introduction, the level of proline-hydroxylation can affect the thermal properties of collagen [28], and a correlation between the proline (Pro) and the hydroxyproline (Hyp) percentage in collagen and the habitat temperature has been observed too [29]. Furthermore, lysine (Lys) and hydroxylysine (Hyl) levels are known to be crucial sites in the formation of covalent cross-links along the collagen molecule [20,21], being strictly related to the mechanical properties of collagen itself. For this reason, these amino acids have been the focus of the comparison between the total AA composition of 17 °C and 27 °C sponges. In the specific context of this work, the ratio between Hyp/Hyp + Pro in the extract from sponges collected at a lower temperature is unexpectedly similar to the one of those harvested at a higher temperature (Figure 1). On the other hand, a significant difference can be observed between the Hyl/Hyl + Lys value (Figure 1).

The amino acid composition of *C. reniformis*’ collagen has already been shown in previous literature, but there is no evident agreement in the quantitative data: in Table 1 of the work of Heinemann and co-workers, for example, are clearly listed two different aminoacidic profiles of *C. reniformis*’ collagen, with very strong differences (i.e., in that example Gly percentage varies from 30.6 to 18.9), depending on the methodology of fibers extraction [30]. Different geographic areas of sponge collection and different extraction methodologies undoubtedly have relevance. In this already complex scenario, in the present work, we put in evidence the contribution of seasonality. Sponges collected in the same areas (and with similar genetic characteristics within the population) but in different seasons with different seawater temperatures show some differences in the aminoacidic profile. The higher value of Gly in both types of samples compared to previously published data [30] can be ascribed to differences in the extraction procedures and, probably, to the presence of other proteins intimately mixed with the extracted fibrils.

It is known that environmental temperature variation can affect collagen structural organization, in particular its thermal stability [13]. In poikilothermic aquatic organisms, the adaptation to seasonal temperature change determines many post-translational modifications in collagens, specifically the prolyl and lysyl hydroxylation and protein glycosylation. The proline hydroxylation contributes to the stability of the collagen’s triple helix via a stereo-electronic effect and not by a simple inductive effect, as originally hypothesized [31]. Its impact, however, depends on its abundance and its position in the collagenic chain [17]. In the *C. reniformis* case, the presented aminoacidic profile shows the substantial equivalence of the proline hydroxylation level, suggesting that, apparently, this post-translational modification doesn’t have a key role in the thermal stabilization of the collagen molecule. However, without completely characterizing the aminoacidic sequence of the *C. reniformis*’ fibrillar collagen from both a genetic and a proteomic point of view, we cannot exclude that, with the increase in environmental temperature, although the levels of proline hydroxylation do not vary, a different distribution along the sponge collagen polypeptides could occur with some impact on the thermal stability.

**Figure 2:**
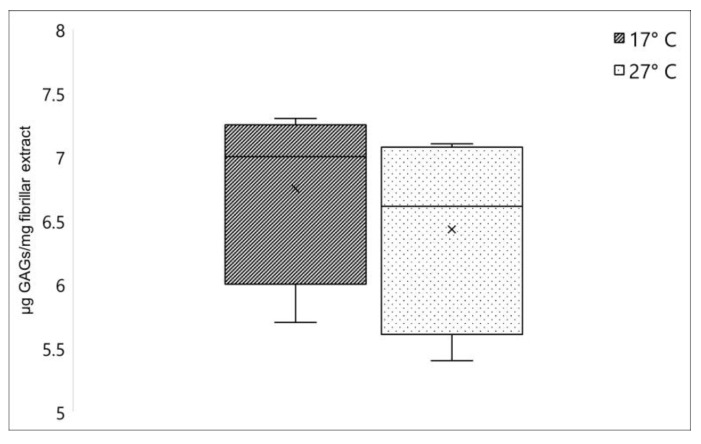
Collagen-associated GAGs amount in *C. reniformis* collected during winter (dark box) and summer (lightbox), expressed as µg GAGs/mg fibrillar extract. ×, mean value.

A different and more clearly explainable situation is observed here for what concerns lysyl-hydroxylation (Table 1 and Figure 2). In this case, the Hyl/Hyl + Lys ratio changes between sponges collected at 17 °C and at 27 °C. In mammalian collagen, lysine is subjected to a different degree of intracellular hydroxylation, depending on collagen type and/or age and tissue distribution. Hydroxylysine can be involved in the glycosylation process intracellularly, and then, when extruded outside, both lysine and hydroxylysine are the targets of further biochemical modification, leading to interchain cross-linking via complex actions of various enzymes—among them, the lysyl oxidases family [21]. Divergences in hydroxylation levels of lysine in collagen extracted from cold and warm periods are indicative of some key role of these amino acids in the different dynamics of *C. reniformis* extracellular matrix related to temperature variation, suggesting a possible relevance in terms of thermal-mechanical properties.

#### 2.1.2. Glycosaminoglycans Quantification

Another relevant component strictly annexed to the collagen fibers in *C. reniformis* fibrillar extract is the glycosaminoglycans (GAGs), which represent one of the major components of the extracellular matrix [3]. No significant differences were registered when comparing the GAGs content between fibrillar extracts isolated from *C. reniformis* collected in winter and in summer. As shown in Figure 2, this polysaccharide fraction resulted in 6.75 ± 0.72 (sd) µg GAGs/mg fibrillar extract and 6.43 ± 0.79 (sd) µg GAGs/mg fibrillar extract for 17 °C and 27 °C samples, respectively.

GAGs are prevalently linked to proteins in the extracellular matrix or, in the case of hyaluronic acid, freely distributed and contribute to the mechanical and rheological properties of the ECM at different levels. GAGs are present at different concentrations and chemical forms in distinct tissues, and their concentration also varies with age or in the occurrence of specific pathologies in humans and mammalians [32,33,34]. Their specific role in sponges is not completely clarified yet, but analogously to other animal phyla, their presence in the extracellular matrix is proved, with a potential involvement also in the cell-matrix interactions mechanisms [35]. Although, these polysaccharide derivatives in *C. reniformis* are intimately connected to collagen fibrils, giving them interesting rheological properties after extraction, with potential biotechnological application [24]. Our data indicate that GAGs cannot play a relevant role in the modifications of collagen properties during seasons, their concentration being independent of temperature changes.

#### 2.1.3. Proteins Glycosylation

**Figure 3:**
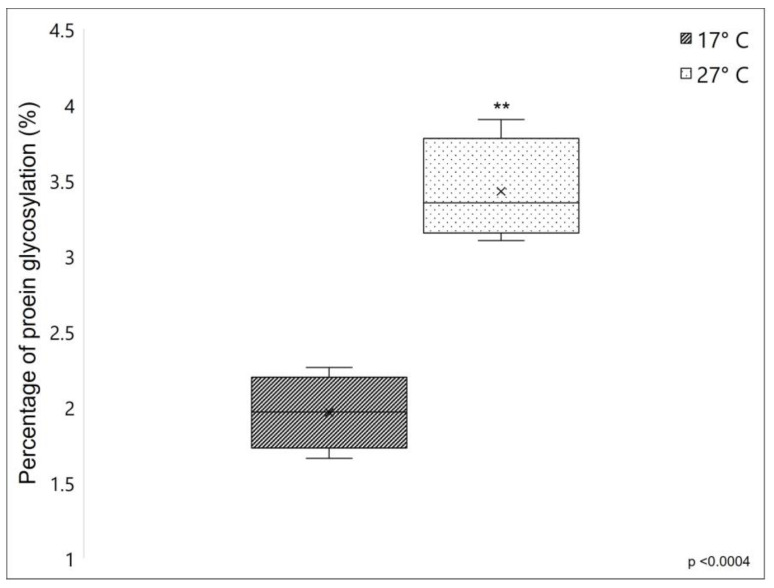
Percentage of glycosylation (%) of collagen extracted from *C. reniformis* in cold (dark box) and warm temperatures (lightbox). Asterisks indicate a significant difference versus the respective control (paired Tukey test, ** *p* < 0.001). ×, mean value.

The percentage of protein glycosylation was compared between fibrillar collagen extracted from 17 °C and 27 °C sponges, which showed a 1.96 ± 0.246% and 3.43 ± 0.34% of protein glycosylation, respectively (see Figure 3). The significantly higher value of the percentage of protein glycosylation of fibrillar collagen extracted from 27 °C sponges, resulting in 75% more glycosylated than that collected from 17 °C sponges, indicates that in *C. reniformis,* the water temperature has a remarkable impact on the overall levels of collagenic protein glycosylation.

In collagens and collagen-like proteins, one of the main targets of glycosylation events is the hydroxylysine residues in the α-helical domain, which can be subjected to further post-translational modifications by sequential steps of O-linked glycosylation producing G-Hyl (galactosyl-hydroxylysine) and GG-Hyl (glucosyl galactosyl-hydroxylysine) [21]. These carbohydrates are located on the surface of the protein, where they play an important role in lateral interactions between collagen triple helices and between collagen molecules and other extracellular matrix components. However, glycosylation can also occur on other amino acids with functional hydroxyl groups, such as serine and threonine [36]. For instance, in the marine polychaete *Riftia pachyptila*, typically found in hydrothermal vents, an unusual presence of galactosylated threonine has already been described [37,38]; in this animal, the high level of collagen glycosylation seems to contribute to the thermal stability of its collagen significantly. It is, therefore, expected that also in our model, the increased glycosylation rate observed in summer samples could improve the thermal stability of the collagen fibers from 27 °C sponges.

### 2.2. Thermal Stability Analysis

**Figure 4:**
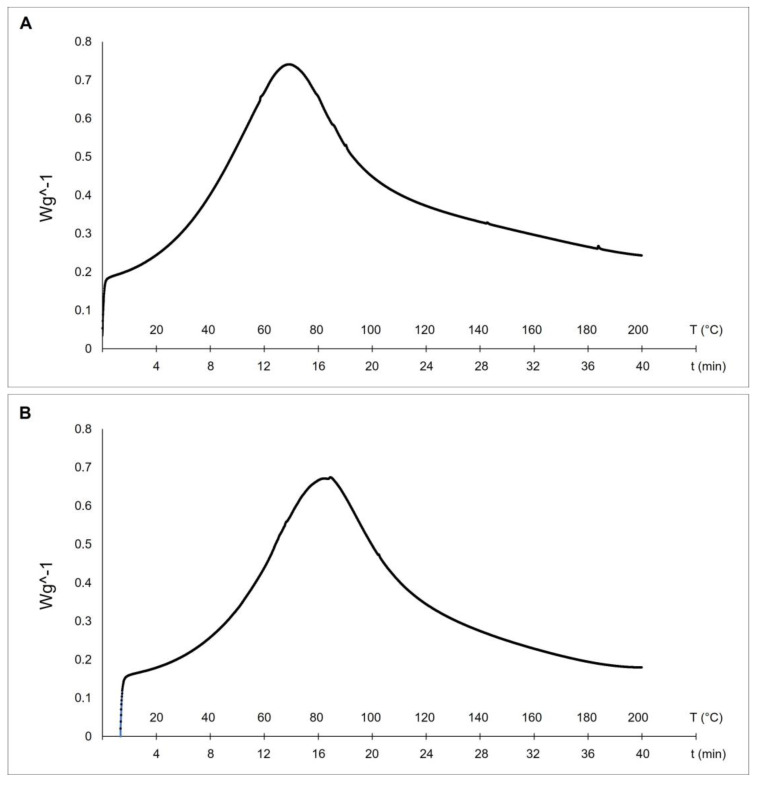
Differential Scanning Calorimetry (DSC) of collagen fibrils extracted from 17 °C sponges, with a melting peak at 69.2 °C (**A**), and 27 °C sponges, with a melting peak at 82.1 °C (**B**).

Differential scanning calorimetry (DSC) is a technique used to evaluate the thermal stability of collagen fibers. The stability of the triple helix in collagenic structures is related to hydrogen bonds. The thermal denaturation of collagen depends on its water content, degree of cross-linking, glycosylation level, and pH of the environmental medium [17]. Our results evidenced higher thermal stability of collagen from sponges collected at 27 °C (with a melting peak at 82.1 °C—see Figure 4B) if compared to the collagens extracted from sponges collected at 17 °C (with a mean denaturation peak at 69.2 °C—see Figure 4A).

These differences can be explained by the documented temperature dependence of collagen thermal stability in animals living at different temperatures [13]. The endothermic peaks of the two temperatures-*C. reniformis*’ collagens are similar to the previously described results from collagen extract isolated from the marine sponge *Ircinia oros* (71.19 °C, [4]), while both are remarkably higher concerning the ones of *Sarcotragus foetidus* (55.79 °C, [4]) as well as to other mammalian collagens (54.17 °C, [4]). In those cases, it was put into evidence higher thermal stability of *I. oros* fibers due to the presence of a sugar sheet around them and to a possible higher level of fibrils cross-linkage. The thermal stability of collagens is directly related to the proline hydroxylation level [17]. Indeed, hydroxyproline has a stabilizing effect because the hydroxyl group of Hyp acts primarily through stereoelectronic effects [39], but in many situations, the presence of Hyp is not the prevalent cause of thermal stability. In *R. pachyptila*, as described above, the major contributor to collagen stability is given by unusual glycosylation sites of the fibrils [37,38] and not by high Hyp levels. In the current study, the Hyp level in collagen extracted from 27 °C sponges was not significantly different from that of 17 °C sponges. This evidence would seem to contrast with the thermal stability trend if this physical-chemical property was exclusively related to the Hyp level. In this case, we can assume that the great differences in the glycosylation percentage of the collagenic proteins could be one of the main contributors to their higher thermal stability, while the Hyp level could play a minor role, accordingly to the peculiar case of *R. pachyptila* [36].

### 2.3. Viscosity Evaluation

**Figure 5:**
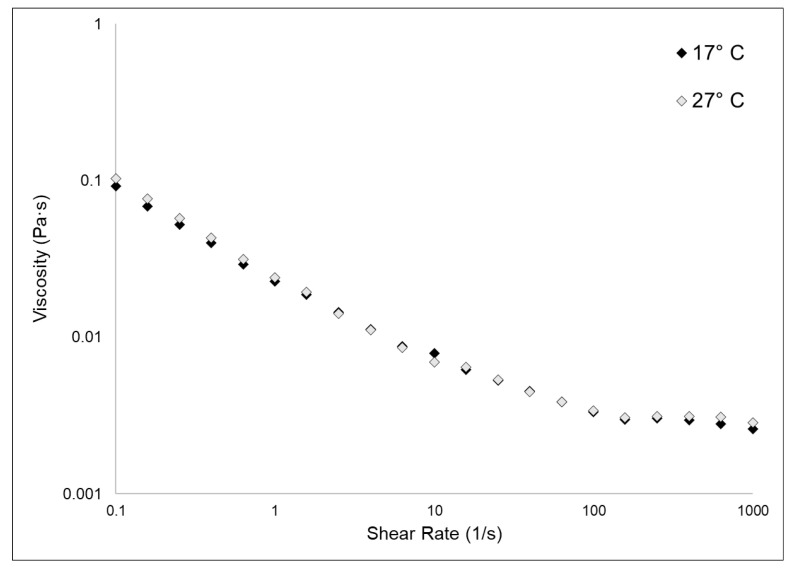
Fibrillar suspensions viscosity test. The graph shows the flow sweep curves obtained in the viscosity tests of the 17 °C and the 27 °C sponges collagenous fibrillar extract by rheological measurements, conducted as explained in the methods section (Section 3.4.5). Curves were fitted by the Carreau–Gahleitner model [40] following the equation: (*η* − *η*_∞_/*η*_0_ − *η*_∞_) = 1/(1 + (a × γ)^b^)^P^ where *η* is the shear viscosity, *η*_∞_ is the infinity-shear viscosity, *η*_0_ is the zero-shear viscosity, a is the Carreau constant, b is the Gahleitner exponent, and P is the Carreau exponent. Table 2 summarizes the experimental values of *η*_0_ and *η*_∞_.

Figure 5 shows the experimental flow sweep curves obtained for 17 °C and 27 °C fibrillar suspensions. Data were fitted by the Carreau–Gahleitner model [40], and the resulting values of η_0_ and η_∞_ are shown in Table 2. As previously observed [3], the fibrillar suspensions showed a low viscosity and were characterized by a shear-dependent viscosity (i.e., η quickly decreases with the increasing of the shear rate). The two different types of samples shared almost identical values of η_0_ and η_∞_. In this kind of biomaterial, the rheological properties are, beyond other factors, related to the glycosaminoglycan content [23]. Our experimental observations showed that the GAGs content in the two samples does not vary, as explained in Section 2.1.2; therefore, it was expected that also their viscosity measurements would not differ from each other.

### 2.4. Collagen Membranes Characterization

The extracted collagen was used to produce 2-D membranes accordingly to the described procedure (see Section 3). To verify if the molecular differences of collagen extracted in winter and in summer could affect the membrane properties, we evaluated the texture, dynamic-mechanical properties, antioxidant potential, and bio-compatibility of the membranes with cell lines.

#### 2.4.1. Membranes Surface Morphologies

**Figure 6:**
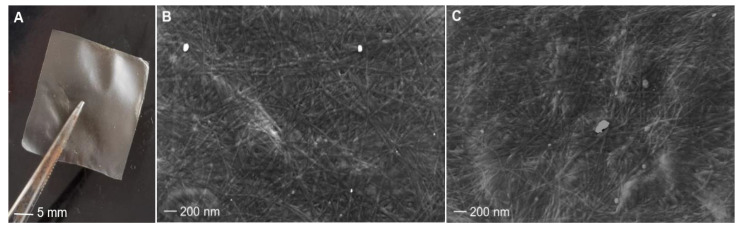
*C. reniformis* collagen membranes and their morphologies. On the left: magnification of one membrane (**A**). In the following images, the ultrastructure of membranes was obtained from 17 °C sponges (**B**) and 27 °C sponges (**C**).

Collagen extracted from sponges collected at 17 °C and 27 °C was cast and dried in silicone molds so that it was possible to recover thin, light membranes that were smooth to the touch (Figure 6A). ESEM analysis showed that both membranes, obtained from cold and warm sponges, have a similar pattern of fiber distribution as well as a similar fiber diameter of about 20 nm ± 0.3 (sd), on average (Figure 6B,C). Although aquaculture experiments assessed an improved growth rate of *C. reniformis* at 25 °C than at lower temperatures [27], our observations indicate that environmental temperature does not distinctly affect the diameter of the collagen fibers of this animal.

#### 2.4.2. Dynamic Mechanical and Thermal Analysis

**Figure 7:**
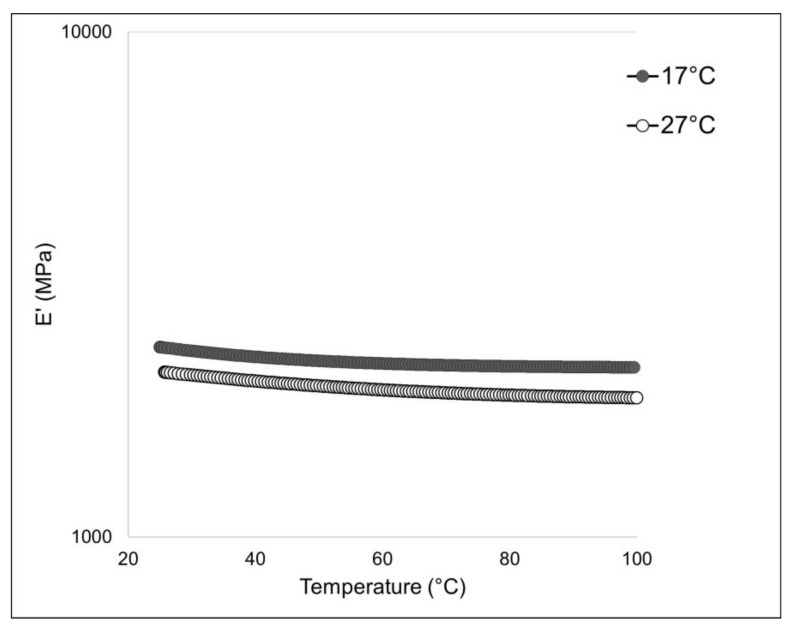
Mechanical analysis of 17 °C and 27 °C sponge-derived membranes: DMTA profile of 17 °C-derived (black curve) and 27 °C-derived (white curve) membranes measured in extensional configuration with a frequency of 1 Hz and an extensional stress of 0.1 MPa.

To assess the mechanical stability of the 2-D membranes, a dynamic-mechanical (DMA) and a dynamic-mechanical-thermal analysis (DMTA) were performed. The outcome of the DMTA test is shown in Figure 7, while the experimental results of the DMA analysis are summarized in Table 3. The membranes obtained with collagen extracted from sponges living at 17 °C display a higher stiffness (i.e., higher elastic modulus E’) if compared to the same membranes produced instead with collagen extracted from sponges living at 27 °C. The collagen extracted from 17 °C sponges could have some inter-chain cross-links that significantly impact the mechanical properties, but, in the meantime, it has a minor relevance on thermal stability compared to the sugar contribution. On the other hand, the glycosylation level seems to have no relevant influence on this physical feature. A similar behavior was noticed in the case of the 2D membrane obtained from collagen fibers derived from the marine sponges *S. foetidus* and *I. oros* [4], where the membranes characterized by higher thermal stability had lower stiffness; however, in that context, the compared membranes were made of collagen fibrils of very different calibers, and the differences in the mechanical properties were also attributed to the different textures. In this study, since the texture remained unchanged (Figure 6B,C), the differences in the mechanical properties of the membranes can be ascribed only to a chemical difference. The mechanical properties of collagen fibers are driven by both cross-link density and type/straight [41]. The maturation of enzymatic cross-links enhances the mechanical capabilities of the fibrils. During tissue growth, the collagen present in ECM is first composed of “immature” cross-link (i.e., more labile and unstable cross-links) due to the high turnover of collagen molecules that, during the growth stationary phase, are then stabilized with more stable trivalent cross-links [42]. As previously reported [27], whereas, at higher temperatures, an increased sponge growth rate was observed, our experimental results on the mechanical properties of the sponge collagen suggest that the fibers could, in summer, be characterized by immature cross-links due to the higher molecular turnover. From an ecological point of view, the increased flexibility of the collagen fibers detected in summer could help the peculiar asexual reproduction process of this sponge species, which relies on the creeping of mesohyl, which preferably occurs in this season [33,43].

#### 2.4.3. Membranes’ Antioxidant Properties

**Figure 8:**
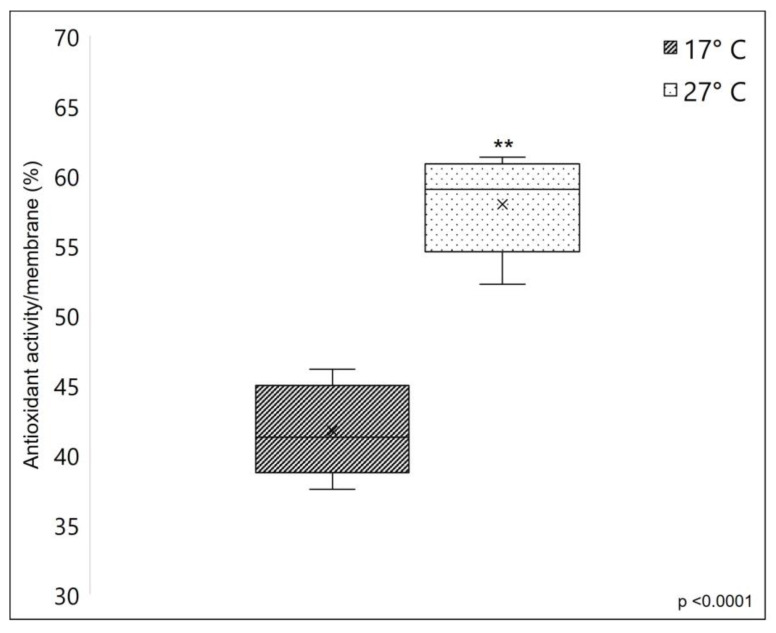
Free radical scavenging activity of the sponge-derived membranes, based on DPPH assay. The scavenging capacity of the SCMs was evaluated as the inhibition percentage of DPPH radical (see Section 3). In this case, the radical scavenging activity was expressed in the function of the membrane surface. The dark box represents the scavenging potential of 17 °C collagen-derived membranes, while the lightbox represents the scavenging potential of 27 °C collagen-derived membranes. Asterisks indicate a significant difference versus the respective control (paired Tukey test, ** *p* < 0.001). ×, mean value.

A remarkable difference was observed in the membranes’ antioxidant properties. Figure 8 clearly shows that membranes obtained from warm sponges have a significantly higher radical scavenging potential (with 38.91% more inhibition, average) compared to membranes obtained from cold sponges, measured using DPPH assay. The strong antioxidant properties of *C. reniformis*’ collagen were already demonstrated by previous works (see [3,23]), where the DPPH scavenging activity was also assayed on collagenic peptide fractions. Here we give additional information, which is that the collagen from 27 °C sponges has remarkably higher antioxidant properties when compared to the same material obtained from sponges grown at 17 °C. The range of scavenging activity of *C. reniformis* membranes obtained from the collagen of sponges collected at warm temperatures is higher than other marine collagens (see [23]) and similar to the collagen from the swimming bladder of some fish species (see [44]). The protocol of collagen extraction and membrane preparation here used seems to maintain this important chemical property, and the analysis of the biochemical characteristics of the two different types of collagen seems to be ascribed to the different glycosylation levels of the 17 °C and 27 °C *C. reniformis*’ collagens. Collagen glycosylation is suggested as one possible contributor to antioxidant activity [23], together with amino acids and other organic components involved in the Maillard reaction [45].

#### 2.4.4. Biocompatibility Evaluation

**Figure 9:**
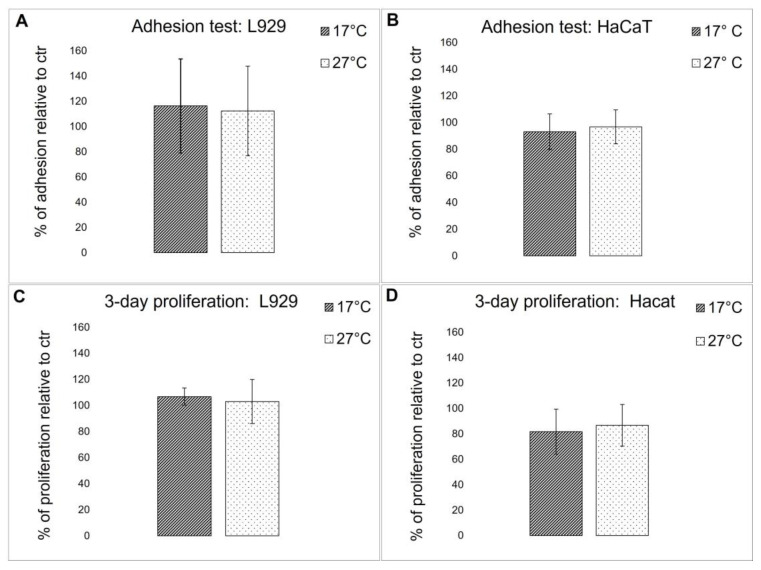
Cell test on collagenic membranes. (**A**,**B**) Cell adhesion quantitative evaluation, by MTT test, of L929 fibroblasts (**A**) and HaCaT keratinocytes (**B**) on collagen from 17 °C sponges (dark bars) and collagen from 27 °C sponges (light bars) pre-coated plates after 18 h of incubation. The results are expressed as cell percentages with respect to controls (rat tail type I collagen) and are the mean ± S.D. of three experiments performed using eight wells for each experimental condition. (**C**,**D**) Cell viability quantitative evaluation, by MTT test, of L929 fibroblasts (**C**) and HaCaT keratinocytes (**D**) on collagen from 17 °C sponges (dark bars) and collagen from 27 °C sponges (white bars) pre-coated plates after 18 h of incubation. The results are expressed as cell percentages compared to the controls (rat tail type I collagen) and are the mean ± S.D. of three experiments performed using eight wells for each experimental condition.

To evaluate if the biochemical difference detected in the fibrillar collagen extract derived from 17 °C and 27 °C sponges could affect the biocompatibility of the 2D-derived membranes, both cell adhesion properties and viability were tested using immortalized human keratinocytes (HaCaT) and L929 adherent mouse fibroblast cell lines. Cell adhesion and viability were compared to control cells that were grown onto rat tail collagen-coated wells. As shown in Figure 9A,B, both cell lines displayed a similar adhesion behavior in both membranes and controls, and no significant differences in cell viability were observed (Figure 9C,D). These results demonstrate that collagen extracted from both warm and cold sponges, although showing some biochemical differences, does not significantly affect their biocompatibility. In [3], it was noted that when the same collagen extract was subjected to EDC/NHS cross-linking, after 3 days, a slight but significant increase in cell proliferation was observed for both L929 and HaCaT keratinocytes. This observation suggests that the cross-link reaction could modify some lateral groups of sponge collagen, improving the overall biocompatibility of the scaffolds.

## 3. Materials and Methods

### 3.1. Chemicals

Unless otherwise specified, reagents were all acquired from Sigma-Aldrich (Milan, Italy).

### 3.2. Sponge Sampling

**Figure 10:**
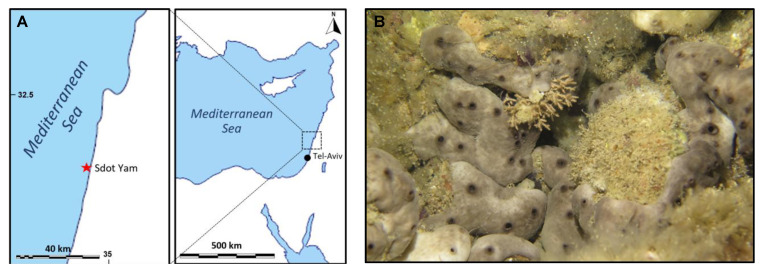
Site of collection of sponges (**A**); a specimen of *C. reniformis* in the collection site (**B**).

Sponges have been collected in the eastern Mediterranean (Sdot-Yam coast, Israel, Figure 10A) in two different seasons: summer (19 July) and winter (17 December). Several specimens of *Chondrosia reniformis* (six for each condition) were harvested by scuba-diving from shallow Israelian waters by the area of the Sdot-Yam coast. The temperature of the water column was measured locally by operators via a Suunto diving computer D4i, registering 17 °C during winter and 27 °C during summer, respectively. The animals were then kept in a cooler until their arrival in the laboratory, where they were frozen whole; then, they were sent to Italy and conserved at −20 °C until used for the analyses and the production of the membrane. The same site of collection allows comparison between similar sponge specimens both from a genetic and an environmental point of view. During summer, sponges are characterized by faster growth [27] and by relevant changes in their body structure and matrix organization if compared to wintertime. In particular, it is known that *C. reniformis* shows a remarkable creeping behavior mainly during summer [33] and, more in general, creeping behavior and mesohyl stiffness are temperature dependent. In the Ligurian sea, for example, the lower the temperature, the stiffer the mesohyl [34,46]. This amazing behavior could be related to some dynamic extracellular matrix biopolymers modifications, and these seasonal changes may, therefore, have an impact on the properties of the extracted collagens for biomaterials production.

### 3.3. Fibrillar Collagen Extraction

Collagen suspensions were obtained from a pool of six specimens for each of the two types of sponge samples, as described in [47], with some modifications. Briefly, portions of tissue of about 1 g each were taken from the frozen animals’ bodies by coring, paying attention to including both the ectosome and the choanosome to be able to consider the natural internal variability of these animals in its entirety.

Sponge tissue was minced into tiny pieces and put into 5 volumes of 100 mM ammonium bicarbonate (pH 8.5), then 0.1% trypsin was added, and the samples were left overnight at 37 °C on a horizontal shaker. Afterward, the liquid part was removed by filtration with a metallic strainer, and the solid material was suspended in 3 volumes of cool deionized water and incubated at 4 °C for 3 days in a rotary disk shaker, aliquoted in 50 mL tubes. The dark and viscous suspension, thus, obtained, was separated from the residual tissue and centrifuged at 1200× *g* for 10 min at 4 °C to remove cell debris and sand particles. The supernatant fluid containing the collagen suspension was finally recovered by centrifugation at 12,000× *g* for 20 min at 4 °C. The obtained pellet was washed twice with 20 mL of deionized water to completely remove any residues of trypsin and resuspended in 10 mL of deionized water. The final fibrillar collagen suspensions were stored at 4 °C until use. To establish the concentration of the two pools of fibrillar collagen extracts, 1 mL of each suspension was lyophilized, and the dry material was weighed.

### 3.4. Biochemical Characterization of Collagen Extracts

#### 3.4.1. Evaluation of Amino-Acids Percentage Composition

Collagen suspensions were subjected to hydrolysis in 2 M NaOH for 20 min at 120 °C at 1 atm in an autoclave and then sent to the Large Instrument Center of the University of Pavia at the Primary Protein Structure Laboratory. Here, the amino acid analysis was conducted using the pre-column derivatization method with OPA (O-Phthal-Aldehyde) and FMOC (9-FluorenylMethyl-Chloroformate) using an HP Amino Quant series II 1090L connected to a Pentium III Jasco X -LC with a fluorescence detector connected to HP ProDesk Core i5.

#### 3.4.2. Glycosaminoglycans (GAGs) Quantification

Quantification of the glycosaminoglycans (GAGs) content of the fibrillar collagen extracts was made for the two types of samples using the Alcian-Blue GAGs assay, as described in [48]. 20 μL of each collagen extract were added to 20 μL of a solution containing 0.027 M H_2_SO_4_, 4 M guanidine-HCl, and 0.375% Triton X-100; then, GAGs were stained with 0.2 mL of a 0.018 M H_2_SO_4_ working dye solution (WDS) containing 0.005% Alcian Blue and 0.25% Triton X-100. All samples were incubated for 10 min at room temperature onto a horizontal shaker and then centrifuged at 18,000× *g* for 10 min at 4 °C. The stained GAGs pellet, thus, obtained was fully solubilized with 0.4 mL of 4 M guanidine-HCl. The absorbance of each sample was read at 620 nm using a Beckman spectrophotometer (DU 640), with a shark cartilage chondroitin-sulfate standard curve used as a comparison. The procedure was performed in triplicate.

#### 3.4.3. Evaluation of the Percentage of Protein Glycosylation

The percentage of protein glycosylation of fibrillar collagen extracts was evaluated with the Glycoprotein Carbohydrate Estimation Kit (Thermo Fisher Scientific, Milan, Italy). Samples were fully solubilized using 8 M urea and normalized to the same concentration of 2.5 mg/mL by diluting them with the Glycoprotein Assay Buffer provided by the kit. As a comparison, a standard curve with proteins having a known percentage of glycosylation, provided by the kit ad diluted 1:1 with 8 M urea as well, was made. Each sample was added with 0.1 mL of a sodium meta-periodate solution, vigorously shaken, and incubated at RT for 10 min; 0.3 mL of Glycoprotein Detection Reagent were then added. Samples were covered and left at RT for 1 h. Finally, sample solutions were read at 550 nm using a Beckman spectrophotometer (DU 640). The percentage of glycosylation of the samples was obtained by interpolation of the standard curve. The procedure was performed in triplicate.

#### 3.4.4. Differential Scanning Calorimetry (DSC) Analysis

At least 3 mg of fibrillar collagen suspensions derived from 17 °C sponges or 27 °C sponges were left to dry at room temperature directly into aluminum crucibles. DSC analyses were performed with a DSC1 STARe System (Mettler-Toledo, Greifensee, Switzerland). Samples were analyzed at an increasing temperature from 0 to 200 °C, with a heating rate of 5 °C/min. During the DSC runs, a nitrogen flow at a rate of 20 mL/min was constantly applied. As a control, 3 mg of a commercial porcine collagen membrane Bio-Gide^®^ (Geist-lich Pharma AG, Wolhusen, Switzerland) was used and analyzed as described above.

#### 3.4.5. Viscosity Evaluation

The rheological measurements were performed with an Anton Paar Physica MCR 301 Rheometer (Anton Paar, GmbH, Graz, Austria), which was equipped with a 50 mm cone/plate geometry (CP50). The viscosity curves were carried out using a shear rate range between 0.1 and 1000 s^−1^, and each sample was tested twice to check for repeatability. The Rheometer was used with a Peltier heating system for accurate control of the temperature. All of the measurements were performed at 20 °C.

### 3.5. 2D Sponge Collagen Membranes Production

2D rectangular sponge collagen membranes (SCM) were obtained by casting a 2 mg/mL fibrillar collagen suspension derived from each sample type, as previously described in [3]. 3.3 mL of the above-mentioned suspensions were put into 25 × 28 mm silicone molds for DPPH Radical Scavenging Activity analysis, while 2.5 mL were put into 10 × 45 mm plastic molds for DMA/DMTA tests; both were left to dry overnight at 37 °C, resulting in thin sponge collagen sheets.

### 3.6. Sponge Collagen Membranes Characterization

#### 3.6.1. Field-Emission Scanning Electron Microscope (FE-SEM) Observation

To evaluate any changes in fibril diameter and/or pore areas in the two types of membranes obtained, an FE-SEM observation was performed. Firstly, SCMs were soaked into a series of alcoholic solutions with an increasing concentration of ethanol up to 100% to dehydrate them completely; then, they were covered with graphite and examined. The images of the two types of SCMs were observed and acquired with an FE-SEM Zeiss SUPRA 40 VP (Carl Zeiss AG, Oberkochen, Germany) and its associated software. The fibrillar diameter of the SCMs was analyzed by performing physical measurements on the images of the various membranes acquired with the FE-SEM, using the ImageJ free software (Rasband, W.S., ImageJ, U. S. National Institutes of Health, Bethesda, MD, USA, 1997–2016, https://imagej.nih.gov/ij/, 19 March 2023). Means ± S.D. were calculated on at least 40 random measurements of fibril diameter performed on each sample, as previously described [4].

#### 3.6.2. DPPH Radical Scavenging Activity

For the radical scavenging activity of each type of SCM, 25 × 28 mm membranes (with an area of 700 mm^2^) were firstly dipped into 500 μL of deionized water and then soaked into 250 μL of 0.1 mM DPPH in methanol solution (2,2-diphenyl-1-picrylhydrazyl, Calbiochem^®^, Millipore SpA, Milan, Italy). A negative control sample was prepared in the same way, using deionized water. Samples were incubated in the dark for 30 min at RT. After that, the membranes were removed with tweezers, leaving the sample solutions ready to be read at 517 nm versus a blank sample that was prepared by replacing the DPPH solution with methanol; a Beckman spectrophotometer (DU 640) was used. The scavenging capacity of the SCMs was evaluated as the inhibition percentage of DPPH radical using the following equation:

DPPH radical scavenging activity (%) = (A0 − A)/A0 × 100%(1)

where A was the sample absorbance rate; A0 was the absorbance of the negative control. The procedure was conducted in triplicate.

#### 3.6.3. Dynamic Mechanical Analysis (DMA) and Dynamic Mechanical-Thermal Analysis (DMTA)

The two types of SCMs were subjected to dynamic mechanical (DMA) and dynamic mechanical-thermal analysis (DMTA) using an MCR 301 rheometer (Anton Paar, GmbH, Austria) provided with a universal extensional fixture (UXF) geometry and a CDT-450 chamber, as described in [4]. Rectangular collagen sheets (40 mm × 10 mm) were prepared with a punch cutter starting from the 10 × 45 mm samples. The thickness of each of them was measured via a digital micrometer. To ensure the correct sample loading and result reliability, a static extensional stress (σ_s_) of 2 MPa was applied for all experiments.

The linear viscoelastic region (LVER) of the samples was first evaluated via amplitude sweep tests (AS) at T = 25 ± 1 °C using a frequency (ν) and oscillatory extensional stress (σ) of 1 Hz and in the range 0.01–10 MPa, respectively. Then, frequency sweep tests (FS) were performed at T = 25 ± 1 °C with a fixed σ = 0.1 MPa, varying the frequency between 0.01 and 10 Hz. Finally, temperature sweep tests (TS) were conducted in a temperature range of 25–100 °C with a heating rate of 2 °C/min at a frequency of 1 Hz and a stress of 0.1 MPa.

### 3.7. Adhesion and Viability Tests on Cells

#### 3.7.1. Cell Cultures

The mouse fibroblast L929 cell line was obtained by the National Collection of Type Cultures (NCTC), while the human keratinocyte HaCaT cell line (CLS Cell Lines Service, 300493) was supplied by the Cell Lines Service (GmbH, Eppelheim, Germany). Cell cultures were kept at 37 °C in a humidified, 5% CO_2_ atmosphere, in high glucose Dulbecco’s modified Eagle’s medium (D-MEM) with glutamax (Euroclone, Milan, Italy), supplemented with 10% FBS (Euroclone) and added with penicillin/streptomycin as antibiotics.

#### 3.7.2. Cell Adhesion and Cell Proliferation

50 μL of the sponge fibrillar extract derived from each sample or of rat-tail collagen (used as control) at a final concentration of 2 mg/mL were left to dry on 96-well plates for 18 h at 37 °C, then the coatings were washed twice with 100% ethanol and finally sterilized under UV-light for 30 min. Before seeding the cells, the collagen coatings were pre-incubated for 1 h at 37 °C with 100 μL of complete D-MEM tissue medium to achieve the total rehydration of the scaffold. L929 and HaCaT cell lines were seeded at a density of 10,000 cells/well on 96-well plates. Cells were allowed to adhere for 18 h at 37 °C in the complete medium; the medium was then removed, the adhered cells were washed once with PBS to remove the floating unattached ones, and finally, an MTT test (0.5 mg/mL final concentration) was conducted to estimate the number of attached cells, comparing the two collagens between each other and to control cells on rat-tail collagen coated wells. Data are means ± S.D. of three independent experiments. To evaluate cell proliferation, experiments were performed on 96-well plates. Both cell lines were plated at a density of 5000 cells/well on pre-coated wells and subsequently cultured for 3 days at 37 °C in a complete medium. At the end of the experiments, once again, an MTT test was performed to evaluate cell viability.

### 3.8. Statistical Analyses

Statistical analyses were performed using one-way ANOVA plus Tukey’s post-test (GraphPad Software, Inc., San Diego, CA, USA). *p* values < 0.05 were considered to be significant.

## 4. Conclusions

In the marine sponge *C. reniformis*, seasonality seems to cause some relevant changes in the extracellular matrix structure and composition, thus, influencing the chemical-physical characteristics of biomaterials produced with the collagen extracted from this animal. More specifically, collagen produced by this sponge during wintertime has some differences compared to the collagen produced in summer. During summertime, is noticeable a slight increase in the Hyl/Lys + Hyl ratio and a remarkable increase in the glycosylation level of the collagen extract. In addition, collagen fibers isolated from *C. reniformis* sponges sampled in summer at 27 °C display higher thermal stability than those isolated from sponges sampled in winter at 17 °C.

The higher thermal stability could be due to an increased level of lysine hydroxylation and an increased level of collagen glycosylation. Conversely, membranes obtained with collagen extracted from 17 °C sponges showed higher stiffness than 27 °C collagen, presumably because of the less mature collagen fibrils present in the actively growing sponges. The seasonal biochemical variations observed in the *C. reniformis*’ fibrillar collagen extracts do not affect the biocompatibility of the membranes, while the increased protein glycosylation in the samples at 27 °C improves their antioxidant properties.

While designing collagen-based biomaterials, researchers should consider the seasonal modifications of *C. reniformis* collagenous extracts observed in this study: sponges from higher temperatures provides collagen with higher thermal stability and greater antioxidant capacity, while sponges from lower temperatures give more mechanically stable collagen. Thus, it will be possible to select the most suitable sponge-collecting (or culturing) temperature based on the specific applications for which this biomaterial is intended.

## Figures and Tables

**Table 1:** Amino acid composition of fibrillar extract from *C. reniformis* collected at 17 °C and 27 °C (per 100 residues).

AA	17 °C	S.D.	27 °C	S.D.
	Mean (%)		Mean (%)	
Asp	7.56	0.09	7.47	0.24
Glu	7.34	0.15	7.24	0.16
Ser	4.76	0.58	4.71	0.36
Gly	46.59	2.33	49.48	2.43
Thr	2.43	0.17	2.08	0.18
Ala + Arg	7.68	0.15	7.91	0.19
Tyr	0.97	0.20	0.80	0.08
Val	1.27	0.17	1.04	0.12
Met	1.04	0.35	0.78	0.07
Hyl	2.40	0.31	2.13	0.15
Phe	1.41	0.17	1.26	0.09
Ile	0.99	0.17	0.82	0.08
Leu	2.05	0.14	1.90	0.11
Lys	2.04	0.28	1.71	0.14
Hyp	5.98	0.06	5.51	0.19
Pro	5.50	0.13	5.14	0.26

**Table 2:** Rheological measurements values of η_0_ and η_∞_ of the two fibrillar extracts from which were built the flow sweep curves showed in Figure 5.

Sample	*η*_0_ (mPa·s)	*η*_∞_ (mPa·s)
17 °C	92.23	2.58
27 °C	103.01	2.84

**Table 3:** Mechanical analysis of 17 °C and 27 °C sponge derived membranes: experimental results of DMA test conducted at 25 °C and 37 °C.

	17 °C	S.D.	27 °C	S.D.
E’ @ 1 Hz, 25 °C (MPa)	2368.84	73.05	2105.30	45.78
E” @ 1 Hz, 25 °C (MPa)	181.64	33.61	138.59	34.22
E’ @ 1 Hz, 37 °C (MPa)	2156.03	53.25	1864.24	28.99
E” @ 1 Hz, 37 °C (MPa)	90.88	14.22	77.98	4.41

## Data Availability

The original data presented in the study are included in the article; further inquiries can be directed to the corresponding author.
